# Autophagy-Related Gene Pairs Signature for the Prognosis of Hepatocellular Carcinoma

**DOI:** 10.3389/fmolb.2021.670241

**Published:** 2021-05-20

**Authors:** Yiming Luo, Furong Liu, Shenqi Han, Yongqiang Qi, Xinsheng Hu, Chenyang Zhou, Huifang Liang, Zhiwei Zhang

**Affiliations:** ^1^ Hepatic Surgery Center, Tongji Hospital, Tongji Medical College, Huazhong University of Science and Technology, Wuhan, China; ^2^ Hubei Key Laboratory of Hepato-Pancreato-Biliary Diseases, Wuhan, China; ^3^ Key Laboratory of Organ Transplantation, Ministry of Education, NHC Key Laboratory of Organ Transplantation, Key Laboratory of Organ Transplantation, Chinese Academy of Medical Sciences, Wuhan, China

**Keywords:** hepatocellular carcinoma, autophagy-related gene, gene pairs, prognosis signature, nomogram

## Abstract

Hepatocellular carcinoma (HCC) has been recognized as the third leading cause of cancer-related deaths worldwide. There is increasing evidence that the abnormal expression of autophagy-related genes plays an important role in the occurrence and development of HCC. Therefore, the study of autophagy-related genes can further elucidate the genetic drivers of cancer and provide valuable therapeutic targets for clinical treatment. In this study, we used 232 autophagy-related genes extracted from the Human Autophagy Database (HADb) and Molecular Signatures Database (MSigDB) to construct 1884 autophagy-related gene pairs. On this basis, we developed a prognostic model based on autophagy-related gene pairs using least absolute shrinkage and selection operator (LASSO) Cox regression to evaluate the prognosis of patients after liver cancer resection. We then used 845 liver cancer samples from three different databases to test the reliability of the risk signature through survival analysis, receiver operating characteristic (ROC) curve analysis, univariate and multivariate analysis. To further explore the underlying biological mechanisms, we conducted an enrichment analysis of autophagy-related genes. Finally, we combined the signature with independent prognostic factors to construct a nomogram. Based on the autophagy-related gene pair (ARGP) signature, we can divide patients into high- or low-risk groups. Survival analysis and ROC curve analysis verified the validity of the signature (AUC: 0.786—0.828). Multivariate Cox regression showed that the risk score can be used as an independent predictor of the clinical outcomes of liver cancer patients. Notably, this model has a more accurate predictive effect than most prognostic models for hepatocellular carcinoma. Moreover, our model is a powerful supplement to the HCC staging indicator, and a nomogram comprising both indicators can provide a better prognostic effect. Based on pairs of multiple autophagy-related genes, we proposed a prognostic model for predicting the overall survival rate of HCC patients after surgery, which is a promising prognostic indicator. This study confirms the importance of autophagy in the occurrence and development of HCC, and also provides potential biomarkers for targeted treatments.

## Introduction

Hepatocellular carcinoma, the predominant primary tumor of the liver, has been recognized as the third leading cause of cancer-related death worldwide (Forner et al., 2018). Many patients are diagnosed when the cancer has already metastasized and a series of severe complications have occurred, indicating that the liver cancer has reached an advanced stage (Cabibbo et al., 2010). In site of recent advances in surgical resection or liver transplantation, the 5-year survival rate of HCC patients remains relatively low (Bosetti et al., 2014; Singal and El-Serag, 2015). Therefore, extensive analysis is urgently needed to identify reliable prognostic biomarkers and develop therapies that can target the major oncogenes of HCC.

Autophagy is an important intracellular selective recycling mechanism through which cell components are transported to lysosomes for degradation to recover materials and provide energy (Mizushima, 2018). Due to its unique functions, autophagy is closely related to many human diseases, including immune diseases (Gukovskaya et al., 2017; Yang et al., 2017), neurodegenerative diseases (Hu et al., 2017; Moloudizargari et al., 2017) and different types of cancer (White, 2015; Gugnoni et al., 2016). A large number of studies have shown that autophagy has two opposite effects during the occurrence of common cancers, especially in HCC (Czaja et al., 2013). At the same time, there is increasing evidence that abnormal expression of autophagy-related genes plays a pathogenic role in the development of multiple human diseases, including cancer (Mizushima, 2018). As autophagy plays a key role in hepatocellular carcinoma, prognostic signatures based on autophagy-related genes can help us explore the genetic control mechanism of hepatocellular carcinoma and provide valuable therapeutic targets (Lin et al., 2018). However, few studies have used autophagy-related genes to construct prognostic signatures for HCC.

In this study, we developed and validated a promising prognostic model for HCC based on autophagy-related gene pairs. First, we collected sequencing data of autophagy-related genes from three independent groups to screen for candidate gene pairs. We screened out nine gene pairs that are closely related to the patients’ prognosis and used them to construct a gene-pair model. After calculating the risk scores of the patients using the model, we divided the patients into two groups with significant differences in prognosis. In a series of subsequent verifications, our model showed a good prognostic ability for HCC patients. Our promising prognostic model confirms the important role of autophagy in HCC and provides potential therapeutic targets.

## Materials and Methods

### Data Sources

We obtained an RNA-seq dataset (*n* = 377), which was used as a training set to build the model, and the corresponding clinical information of HCC patients from The Cancer Genome Atlas (TCGA) using the UCSC Xena browser (https://xenabrowser.net/). The validation set was based on a second RNA-seq dataset (*n* = 243) downloaded from the International Cancer Genome Consortium (ICGC) (https://dcc.icgc.org) and a microarray dataset (GSE14520, *n* = 225) from GEO database (http://www.ncbi.nlm.nih.gov/geo). We extracted 232 autophagy-related genes from the Human Autophagy Database (HADb, http://www.autophagy.lu/index.html) and 394 from the GO_AUTOPHAGY gene set in the Molecular Signatures Database v7.1 (MSigDB, http://software.broadinstitute.org/gsea/msigdb). Our autophagy-related gene set was formed by the integration of these two gene sets.

### Data Preprocessing

Specimens of HCC patients who survived less than one month or whose clinical data were incomplete were not included. We removed the data of normal tissue samples and only kept the data of the primary tumor. When multiple specimens were taken from the same patient, the average gene expression value was used to represent the patient’s gene expression level. Only the sequencing data of autophagy-related genes were retained. When the same gene was matched by multiple probes, we used the average expression value of multiple probes to indicate the expression level of the gene. For the RNA-seq data from TCGA database, we excluded HCC samples in which more than half of the gene probe expression values were zero. The expression profiles of common autophagy-related genes were screened from the three data sets.

### Establishment of the Prognostic Model Based on Autophagy-Related Genes

We compared the expression values of autophagy-related genes in each sample to obtain the score of each ARGP. The expression values of autophagy-related genes in each sample were compared in pairs to calculate the score of each ARGP. In a pairwise comparison, if the previous value is greater than the next value, the output is 1, and if it is not, the output is 0. We excluded ARGPs that scored 0 or 1 in more than 90% of the samples in each dataset, and the remaining ARGPs were used to establish the prognostic model for HCC. First, we performed univariate Cox regression analysis using the R package “survival” to select gene pairs that are related to the overall survival of HCC patients in TCGA. Differences with *p* < 0.001 were considered statistically significant. To minimize the risk of overfitting, we used “glmnet” R package to conduct LASSO penalized Cox regression (3,000 iterations) to calculate the frequency of the models. The gene pair model with the highest frequency among the iterations was used to establish a prognostic model. Stepwise multivariate Cox regression analysis was performed.

### Validation and Assessment of the Autophagy-Related Gene Pair Signature

After calculating the risk score in every dataset, the patients were classified into high-risk and low-risk groups according to the median value of the risk score. Kaplan–Meier survival analysis (*p* < 0.05) was used to analyze the over survival (OS) of the high-risk and low-risk groups. After drawing ROC curves for 1, 3 and 5 years, we used the area under curve (AUC) value to verify the accuracy and sensitivity of this model. The closer the AUC value is to 1, the better the predictive effect of the prognostic model. To perform multivariate Cox regression analysis, available clinical and pathological data were integrated with the ARGP signature. Tumor stage, grade, age, and sex were regarded as continuous variables. In addition, we selected three representative prognostic gene models for HCC. Our model was compared with these existing models using the 5-year multiple ROC curves. The respective AUC values were used to estimate the prognostic accuracy of each signature.

### Gene Set Enrichment Analysis

In order to further reveal the biological mechanisms through which the identified autophagy-related genes contribute to the development of HCC, we used the MSigDB hallmark gene set (h.all.v7.1.symbols.gmt) to run gene set enrichment analysis (GSEA). We used an FDR value < 0.25, a nominal (NOM) *p* < 0.05, and | NES | > 1 as the screening criteria to identified signaling pathways that are highly related to the model genes.

### Construction of a Nomogram

Independent prognostic factors that are highly correlated with OS in HCC patients (*p* < 0.05) were screened out using univariate and multivariate Cox regression analyses. We then integrated these independent prognostic factors using the R package “RMS” and constructed the predictive nomogram and corresponding calibration diagram for 1, 3, and 5 years. The calibration maps were verified by calibration and discrimination. The expected possibility of collinearity was plotted graphically as an observable indicator to assess the alignment of the nomogram. The closer the calibration curve was to the reference line (diagonal line), the better the predictive effect of the nomogram.

### Statistical Analysis

All statistical analyses were performed using R software (version 3.6.3, https://www.r-project.org/). The OS of the HCC patients in the low- and high-risk groups was compared using the log-rank test, and the Kaplan–Meier survival curves were drawn using the R package “survminer” (version: 0.4.6). The gene pair prognostic signature was established based on the LASSO Cox regression algorithm using the R package “glmnet” (version: 3.0.2). ROC curves and multiple ROC curves were drawn using the R packages “survivalROC” and “timeROC”, respectively.

## Results

### Construction of the Autophagy-Related Gene Pair Signature

After eliminating duplicate genes from HADb and MSigDB, an autophagy-related gene set comprising 527 genes was obtained. As shown in Table 1, this study included 808 HCC patients from three cohorts. ARGPs were constructed using a total of 269 autophagy-related genes that are represented in all three data sets.

**TABLE1:** Clinical and pathologic factors of the datasets used in this study.

	TCGA(n,%)	ICGC (n,%)	GSE14520 (n,%)
Total	346	241	221
Age			
Median age (years)	61	69	50
Mean age (years)	59.44	67.49	50.819
Gender			
Female	110 (31.79%)	61 (25.31%)	30 (13.57%)
Male	236 (68.21%)	180 (74.69%)	191 (86.43%)
TNM stage			
Stage I	163 (47.11%)	36 (14.94%)	93 (42.08%)
Stage II	78 (22.54%)	110 (45.64%)	77 (34.84%)
Stage III	80 (23.12%)	74 (30.71%)	49 (22.17%)
Stage IV	3 (0.87%)	21 (8.71%)	0
NA	22 (6.36%)	0	2 (0.90%)
Grade			
G1	53 (15.32%)		
G2	162 (46.82%)		
G3	114 (32.95%)		
G4	12 (3.47%)		
NA	5 (1.45%)		
Survival status			
Alive	222 (64.16%)	199 (82.57%)	136 (61.54%)
Dead	124 (35.84%)	44 (18.26%)	85 (38.46%)
Median follow-up time (days)	632.5	780	1,569

Abbreviations: TCGA, TCGA LIHC dataset; ICGC, ICGC LIHC dataset;

We removed ARGPs with a score of 0 or 1 in more than 90% of the samples in all datasets, resulting in 1885 ARGPs. We used univariate Cox regression analysis to screen 117 prognostic ARGPs that were significantly associated with overall survival (*p* < 0.001), and established a prognostic gene model of ARGP using Lasso penalty score Cox regression in the TCGA dataset. After multivariate Cox regression analysis, 9 ARGPs were selected to construct the most stable prognostic signatures, and the corresponding coefficients were used to calculate the risk score for our datasets. Details of the 9-ARGP prognostic model are listed in Table 2.

**TABLE 2:** Univariate and multivariate analyses of prognostic factors in terms of OS.

datasets	Variable	Univariate		Multivariate	
		HR (95%CI)	p-value	HR (95%CI)	p-value
TCGA	Risk score (low risk vs high risk)	4.77 (3.46–6.57)	6.16E-24	1.25 (1.19–1.31)	7.98E-18
	Gender (male vs female)	0.75 (0.51–1.1)	0.141,902	0.82 (0.55–1.23)	0.338,054
	Grade (G1 and G2 vs G3 and G4)	1.12 (0.86–1.45)	0.4012	1.16 (0.89–1.51)	0.271,925
	Age (<60 vs ≥60)	1.01 (0.99–1.02)	0.492,155	1.01 (0.99–1.02)	0.228,735
	Stage (I and II vs III and IV)	1.80 (1.46–2.22)	4.72E-08	1.59 (1.27–1.99)	6.14E-05
ICGC	Risk score (low risk vs high risk)	1.11 (1.06–1.16)	1.43E-05	1.11 (1.06–1.17)	4.88E-05
	Gender (male vs female)	0.46 (0.24–0.86)	0.014548	0.34 (0.17–0.65)	0.001113
	Age (<70 vs ≥70)	1 (0.97–1.03)	0.914,617	0.99 (0.96–1.03)	0.764,419
	Stage (I and II vs III and IV)	2 (1.38–2.91)	0.000268	2.15 (1.48–3.13)	5.49E-05
GSE14520	Risk score (low risk vs high risk)	1.05 (1.02–1.09)	0.001776	1.03 (1–1.07)	0.048104
	Gender (male vs female)	1.66 (0.80–3.45)	0.172,844	1.30 (0.62–2.73)	0.487,211
	Age (<50 vs ≥ 50)	0.99 (0.97–1.01)	0.356,607	1.00 (0.97–1.02)	0.741,149
	Stage (I and II vs III and IV)	2.38 (1.78–3.17)	3.40E-09	2.23 (1.66–3.00)	9.76E-08

**FIGURE 1:**
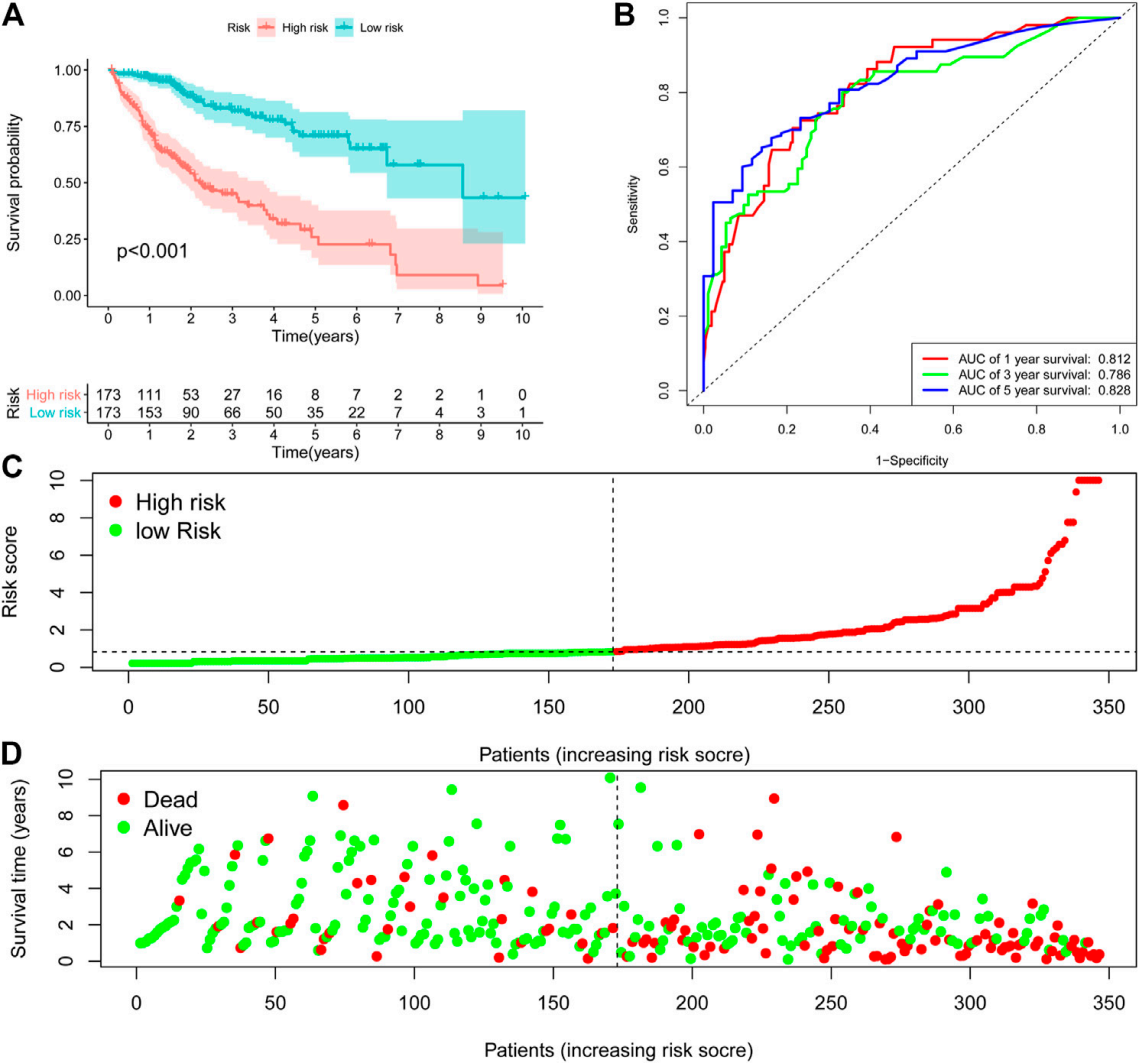
Establishment 9-ARGP signature in the TCGA database. **(A)** Kaplan-Meier survival curves showed the prognostic value of the risk signature between low-risk group (*n* = 263) and high-risk group (*n* = 262). **(B)** ROC curves were used to assess the efficiency of the risk signature for predicting 1-, 3- and 5-y survival. **(C)** The risk scores distribution in the TCGA database. **(D)** The patients survival status in the TCGA database.

The 9-ARGP signature in the TCGA dataset reflected the postoperative prognosis of patients very well (Figure 1). We calculated the risk score of each HCC patient in TCGA according to the prognostic characteristics of autophagy, and then divided the 346 cases into high- and low-risk groups based on the median score. As shown in Figure 1A, the low-risk group had a significantly lower mortality rate than the high-risk group (95%CI: 8.09–21.07, *p* < 0.0001). We evaluated the specificity and sensitivity of the prognostic model using time-dependent ROC curve analysis. The AUC values for 1, 3, and 5 years after surgery reached 0.812, 0.786, and 0.828, respectively, which demonstrated that our ARGP prognostic signature has a promising predictive ability (Figure 1B). The distribution of the autophagy-related prognostic model for patients in the TCGA data set is shown in Figure 1C,D.

### Validation of the Autophagy-Related Gene Pair Signature

**FIGURE 2:**
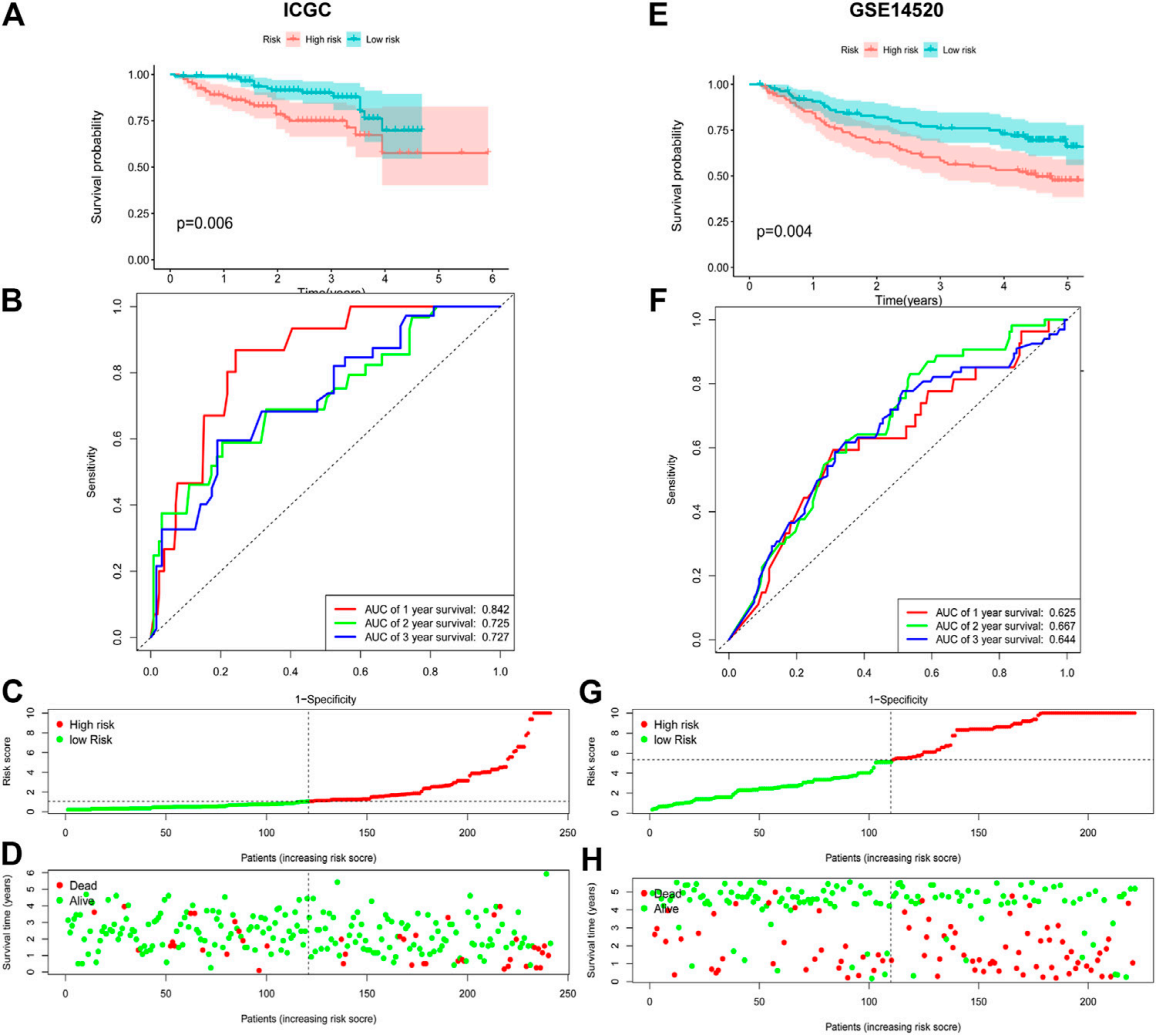
Evaluating the efficiencies of the risk signature in the ICGC and GSE14520 data sets. **(A,E)**, Kaplan-Meier survival curves showed the prognostic value of the risk signature in ICGC data set **(A)**. low-risk group, *n* = 120; high-risk group, *n* = 121; *p* < 0.05) and GSE14520 database **(E)**. low-risk group, *n* = 111; high-risk group, *n* = 110; *p* < 0.001). **(B, F)**, ROC curves evaluated the efficiency of the risk signature for predicting 1-, 2- and 3-y survival in ICGC data set **(B)** and GSE14520 database **(F)**. **(C,G)**, The risk scores distribution in the ICGC data set **(C)** and GSE14520 database **(G)**. **(D,H)**, The patients‘ survival status in the ICGC data set **(D)** and GSE14520 database **(H)**.

In order to further verify its predictive power, we applied the prognostic signature to the ICGC database (containing 243 HCC cases) and the GSE14520 database (containing 225 HCC cases) for analysis. According to the median risk value calculated using the 9-ARGP prognostic signature, HCC patients in the two databases were assigned into high- and low autophagy-based risk groups, respectively. Consistent with the conclusions obtained using the training set, the OS of the high-risk groups in the two validation datasets was significantly lower than that of the low-risk group (*p* < 0.05) (Figure 2A,E). In the ICGC cohort, the AUC values of the prognostic model were 0.842 at 1 year, 0.725 at 2 years, and 0.727 at 3 years (Figure 2B), while in the GSE14520 cohort they were 0.625 at 1 year, 0.667 at 2 years, and 0.644 at 3 years (Figure 2F). Figure 2C,D show the distribution of risk scores corresponding to gene expression levels in the ICGC cohort, while Figure 2G,H shows the corresponding data for the GSE14520 cohort. In univariate Cox regression analysis, TNM staging and the ARGP signature risk score were significantly related to the OS in the three cohorts (HR > 1.00, *p* < 0.05). After correcting for age, gender, grade and TMN staging in multivariate Cox regression analysis, the ARGP signature risk score was still significantly associated with the OS as an independent prognostic factor in the TCGA dataset (HR: 1.25, 95%CI: 1.19–1.31, *p* < 0.0001), ICGC dataset (HR: 1.11, 95%CI: 1.06–1.17, *p*< 0.001) and GSE14520 dataset (HR: 1.03, 95%CI: 1–1.17, *p* = 0.07) (Table 2).

**FIGURE 3:**
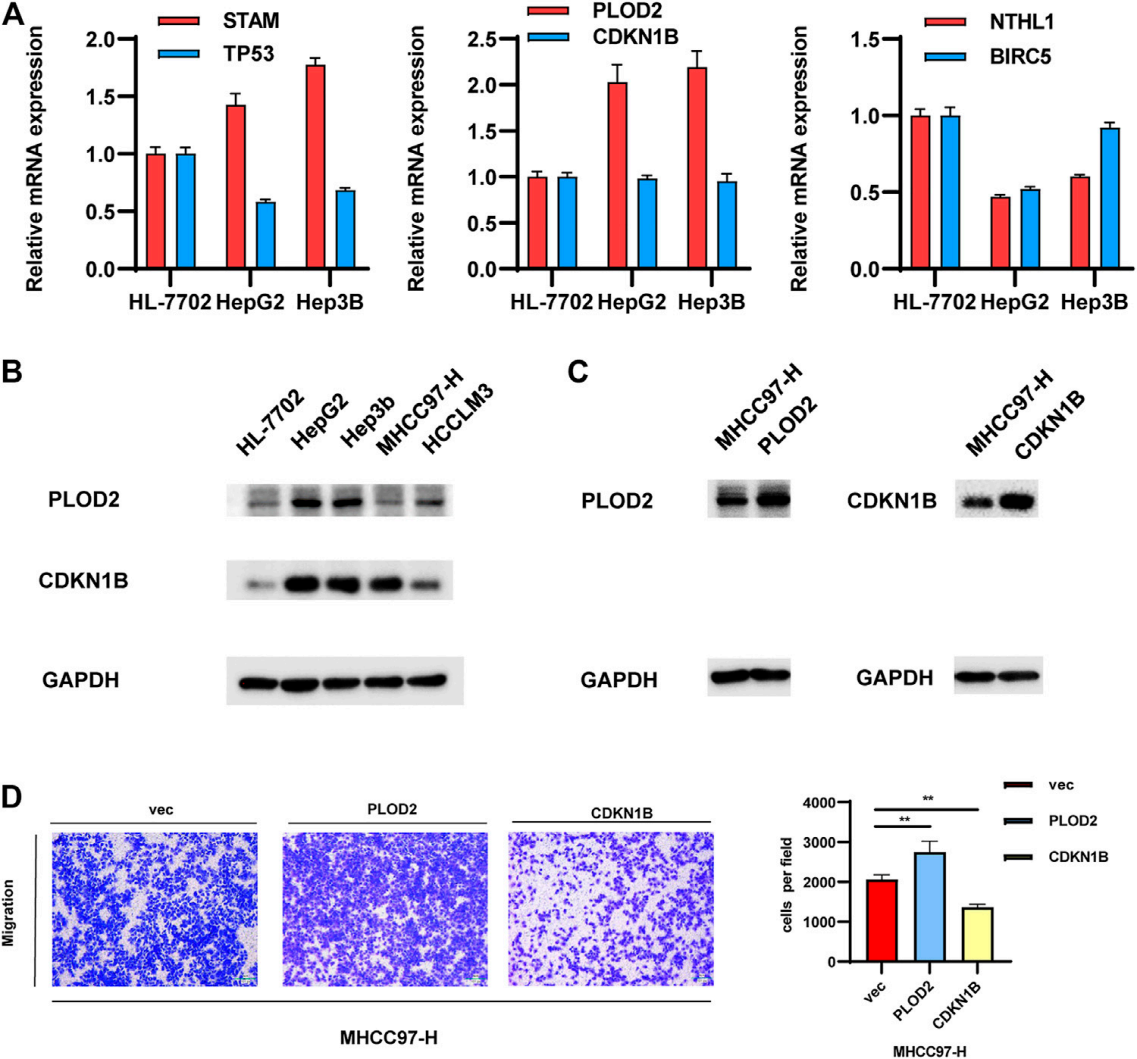
Validation of the gene pairs that make up the model. **(A,B)** Examining the expression levels of representative gene pairs with the large coefficient in normal-liver cell line and in several common HCC cell lines. **(C,D)**. Validate the effects of representative gene pair in HCC cell lines.

The gene pairs with the largest coefficients were STAM/TP53, PLOD2/CDKN1B and NTHL1/BLCR5, since the large coefficients indicate that they have the greatest influence on the model. We examined the expression levels of these genes in a normal liver cell line and in several common HCC cell lines. As shown in Figure 3A, the qPCR results showed that the values of STAM/TP53 and PLOD2/CDKN1B in the HCC cell lines HepG2 and Hep3B were significantly higher than in the normal liver cell line HL7702, which further supported the significance of our model. The coefficient of the gene pair NTHL1/BLCR5 was −0.52, and was significantly lower in the HCC cell lines HepG2 and Hep3B than in the normal liver cell line HL7702, which is also consistent with this conclusion. At the protein level, we found that the expression of PLOD2 in liver cancer cell lines was higher than in normal liver cells, and it was also higher in non-invasive liver cancer cells (Figure 3B).

Further experiments were performed on the representative gene pair Plod2/CDKN1B. We overexpressed these genes in MHCC97-H cells, and the protein levels of PLOD2/CDKN1B were confirmed to be increased after transfection with pcDNA-PLOD2 (oe-PLOD2) and pcDNA-CDKN1B (oe-CDKN1B) (Figure 3C). The results of the transwell assay showed that PLOD2 can promote HCC migration, while CDKN1B had the opposite effect. Since their coefficient is greater than zero, this experimental result was consistent with the previous conclusion (Figure 3D).

We verified the expression levels of the representative gene pair PLOD2 /CDKN1B in 45 liver cancer samples, and combined with the OS and RFS of the corresponding patients, we found that PLOD2 /CDKN1B can better predict the prognosis of the patients. The results are shown in Figure 7. Samples were collected from surgical biopsies of patients who underwent radical resection of liver cancer without preoperative treatment at Tongji Hospital in Wuhan, China, between 2015 and 2018. The Ethics Committee of Wuhan Tongji Hospital authorized this study on patient tissues with written informed consent of the patients.

### Comparison With Representative Published Prognostic Models for Hepatocellular Carcinoma

**FIGURE 4:**
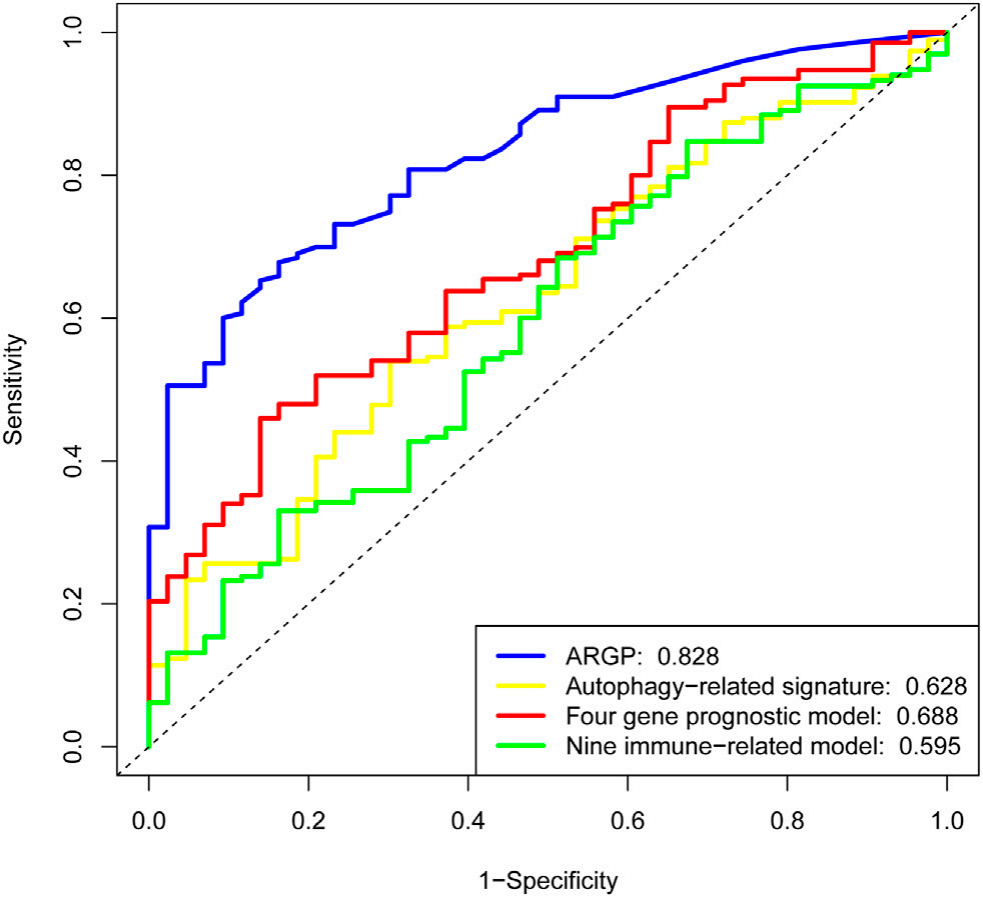
Determination of the receiver operating characteristic (ROC) for different prognostic signatures. The AUC values for the ARGP model, Autophagy-related signature model, four prognostic lncRNA model, and nine immune-related model were 0.828, 0.628, 0.688, and 0.595, respectively. This result indicates that our signature possesses a higher predictive efficacy and accuracy than the other models.

Our ARGP prognostic marker was compared with three published representative gene prognostic markers (Lin et al., 2018; Long et al., 2018; Wang et al., 2020) using ROC curves for 5-year OS. All the data for validation were derived from TCGA. As shown in Figure 4, the AUC value was 0.828 for our prognostic signature, which was obviously more predictive and accurate than the existing autophagy-related signature (AUC = 0.628), the four-gene prognostic model (AUC = 0.688) and the nine immune-related gene model (AUC = 0.595).

### Construction of a Nomogram for Predicting the Over Survival

**FIGURE 5:**
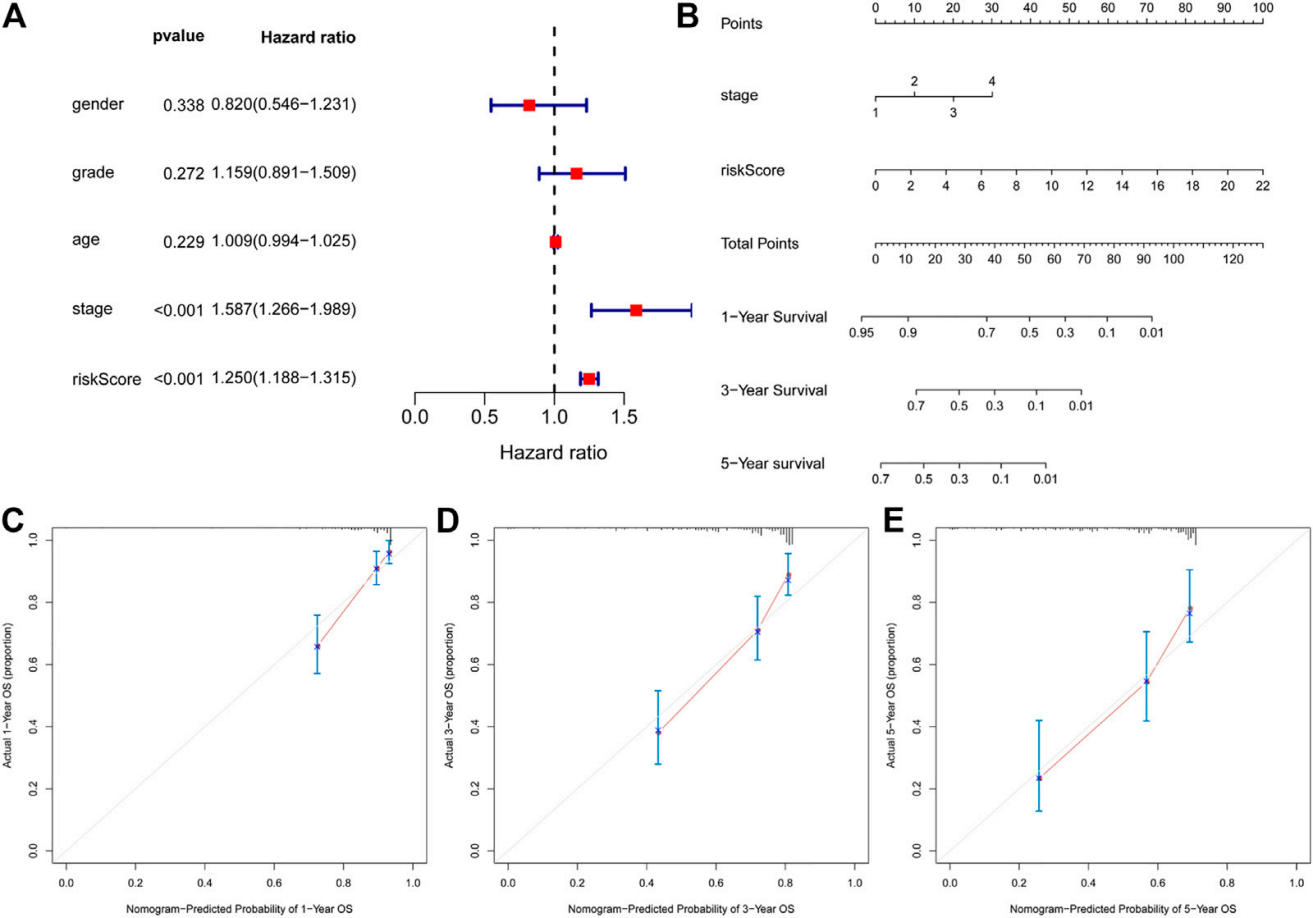
Construction of a nomogram for predicting 1-, 3- and 5-y survival of HCC. **(A)**, Multivariate Cox regression analyses evaluated the contribution of each variable to HCC survival in the TCGA cohort. **(B)** nomogram for predicting 1-, 3- and 5-y survival rate of HCC patients was established. **(C–E)**, Calibration curves showed the probability of 1- **(C)**, 3- **(D)** and 5-y survival **(E)** between the prediction and the observation in the TCGA cohort.

Multivariate Cox regression analysis showed that only TNM stage and the ARGP signature were significant independent prognostic factors for OS (Figure 5A). We attempted to provide a method to more intuitively and accurately predict the survival of HCC patient, which can aid individual clinical decision-making and selection of treatment options. Therefore, a predictive nomogram was constructed based on multivariate Cox regression analysis and combined with two independent prognostic factors (Figure 5B). The scores of each independent prognostic factor were calculated according to the different degree of influence of each independent prognostic factor on the clinical outcomes of the patients, after which the scores were summed up to obtain the total score. Finally, the 1, 3, and 5-year survival rates were predicted based on the functional relationship between the total score and the survival rate. According to the calibration curves of the 1-, 3-, and 5-year nomograms, which were all close to the optimal prediction curve, the predicted OS rate was highly consistent with the actual observed values (Figure 5C–E).

### Physiological Signal Channel Correlated With the Autophagy-Related Gene Pair Model

**FIGURE 6:**
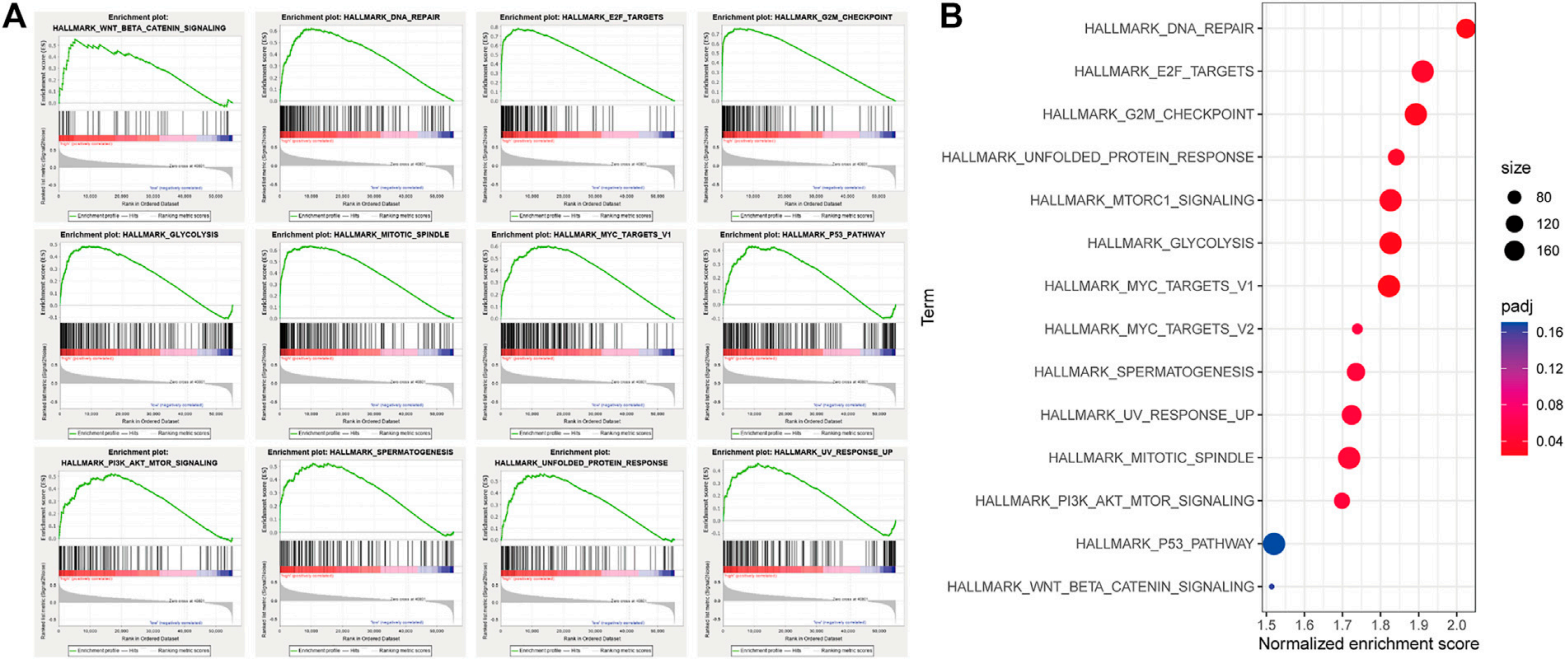
Gene set enrichment analysis (GSEA) between high and low autophagy risk groups. **(A)**, Twelve cancer hallmark gene sets are enriched in the high autophagy risk group in patients with HCC (*p* < 0.05, FDR < 0.25, |NES| > 1). **(B)** bubble chart for visualizing the GSEA result.

We performed GSEA in the high- and low-risk groups from the TCGA cohort, divided according to the median risk value. A total of 12 cancer hallmark gene sets were identified in the high-risk group (Figure 6A). Some of these pathways are “MYC_TARGETS”, “GLYCOLYSIS” and “DNA_REPAIR”, indicating that these signaling pathways are closely related to the progression of HCC. To make the results of enrichment analysis more intuitive, we visualized the significance, the number of included genes and the enrichment score in a bubble chart with different colors, sizes and locations (Figure 6B).

**FIGURE 7:**
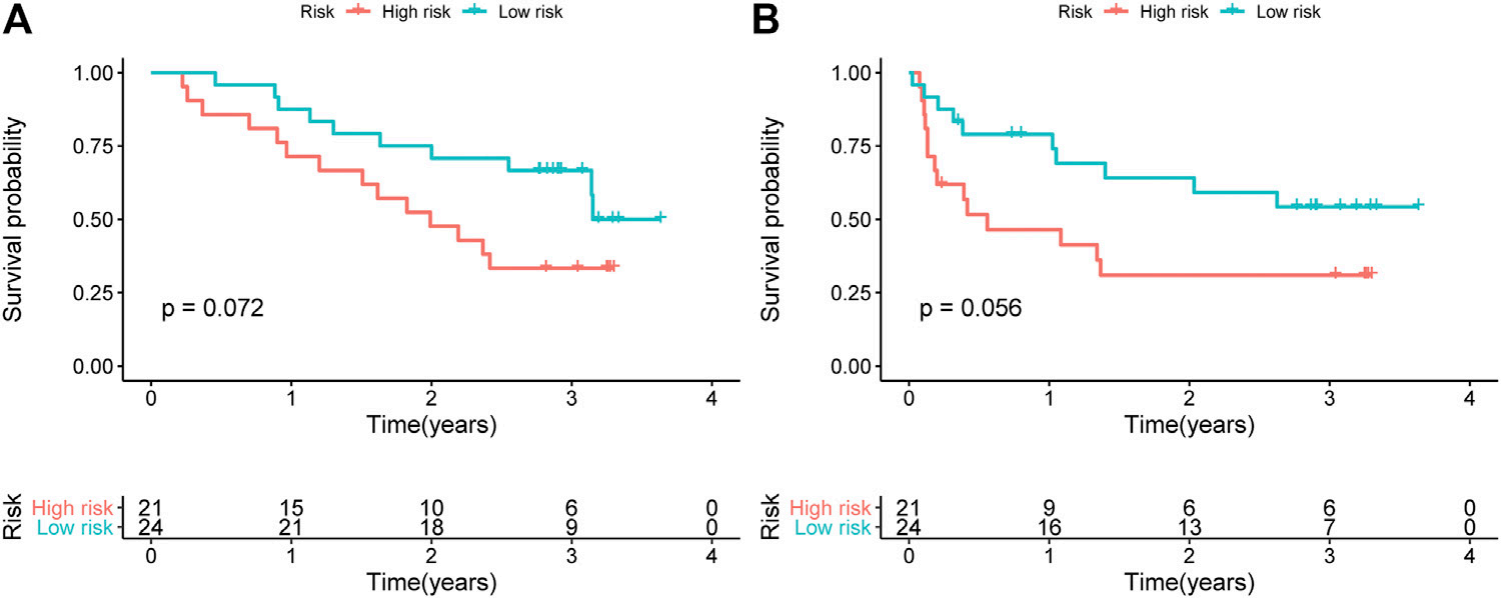
Verified the expression level of the representative PLOD2/CDKN1B in 45 liver cancer samples. Kaplan-Meier curves of **(A)** OS and **(B)** RFS for HCC patients stratified by the signature risk groups in the Tongji cohorts.

## Discussion

Although many environmental or genetic risk factors associated with the occurrence of HCC have been elucidated, the molecular mechanisms underlying the metastasis and recurrence of HCC remain unclear. Consequently, hepatocellular carcinoma remains one of the deadliest malignancies in the world, with exceptionally high recurrence and low survival. In recent years, the application of high-throughput technology and the emergence of large-scale cancer gene expression databases have deepened our understanding of the characteristics of liver cancer and provided the possibility for us to predict postoperative survival rates based on the genetic phenotypes of the individual tumor. Based on gene expression profiles, some studies have established prognostic markers for predicting the survival after liver cancer surgery, while others have explored molecular subtypes of liver cancer based on multi-group analysis (Liu et al., 2020). However, these results are far from clinical application. Due to the diversity of data types among different databases, gene expression levels of different sequencing platforms need to be appropriately standardized before use, but it is still difficult to completely overcome biological heterogeneity and eliminate the technical bias of cross-sequencing platforms. Thus, improving the genetic models and selecting stable specific prognostic markers is still the main task of current liver cancer research.

In this study, we established a prognostic model for HCC based on 9 autophagy-related gene pairs (ARGPs) and validated it across different platforms using the independent datasets ICGC and GSE14520. Our 9-ARGP signature proved to be a significant and excellent predictor in a series of validation analyses, successfully dividing patients into high and low-risk groups with significantly different prognostic outcomes. Compared with three other existing prognostic models for hepatocellular carcinoma, our model showed a more accurate predictive power. We further combined the model with selected significant pathological features. According to the results, the 9-ARGP signature is a powerful complement to HCC staging indicators, and their combination provides a better prognostic performance.

ARGPs were generated based on the pairwise comparison of gene pairs, so there is no need to consider batch differences among different databases. Furthermore, the correlation coefficients of ARGP were calculated based on the gene expression in the same sample, so the data does not need to be standardized. Therefore, our prognostic signature can be used for the individualized prediction of postoperative survival in HCC patients, and it can be more easily applied in clinical practice.

Autophagy has been reported to play a key role in promoting the formation of liver cancer (Czaja et al., 2013). Our 9-ARGP signature contains 17 autophagy-related genes in total. These genes are directly or indirectly related to the occurrence and prognosis of HCC, which has been described in many studies. To provide theoretical support for this statistical signature, we further explored the genes included in the model. The following studies support a mechanistic link between our model and HCC.

CDKN1A/p21 and CDKN1B/p27 are members of a family of cyclin-dependent kinase inhibitors that act as tumor suppressors and inhibit cell proliferation. CDKN1A expression is strictly controlled by the tumor suppressor protein p53 (TP53), which mediates G1 phase arrest in response to various stress factors. CDKN1A can be considered as an independent factor for the development of liver cancer, and in patients with cirrhosis, high expression of CDKN1A may be associated with the occurrence of liver cancer (Wagayama et al., 2002). However, CDKN1A expression is beneficial in patients after hepatectomy and may be an independent prognostic factor for patient survival (Kao et al., 2007). Interruption of the P53-CDKN1A cell cycle pathway may lead to further tumor progression (Lee et al., 2004). The activation of CDKN1A gene expression induced by RNA may have a significant potential for the treatment of HCC and other cancers (Wu et al., 2011). In addition, the subcellular localization of CDKN1A was found to contribute to the development of HCC (Qiu et al., 2011). CDKN1B shares a limited similarity with CDKN1A. Furthermore, reduced CDKN1B expression often predicts poor clinical outcomes in HCC (Huang et al., 2011; Matsuda et al., 2013), and CDKN1B silencing increases the viability of HCC cells (Xu et al., 2019). This is consistent with previous findings that CDKN1B potentially plays an active role as a negative regulator in the early stages of HCC progression (Ito et al., 1999). The risk of HCC is increased by CDKN1A polymorphisms, alone or in combination with CDKN1B polymorphisms (Liu et al., 2013). These studies indicate that both CDKN1A and CDKN1B are closely related with the occurrence of liver cancer and can be used as prognostic biomarkers. BIRC5, a member of the inhibitor of apoptosis (IAP) gene family, promotes cancer development by inhibiting the apoptosis of HCC cells (Zhang et al., 2014), promoting cell proliferation (Sun et al., 2013), enhancing chemoradiotherapy resistance (Liu et al., 2013b) and inducing stromal angiogenesis in the tumor (Fernandez et al., 2014). Similarly, BIRC5 was reported to be directly associated with autophagosome formation and contribute to the survival of HCC cells (Chang et al., 2014). DLC-1 is a GTPase-activating protein that targets Rho (Kim et al., 2007), and as a tumor suppressor, DLC-1 is not only involved in hepatocarcinogenesis, but also inhibits the cancer progression and oncogenic autophagy of hepatocellular carcinoma (Wu et al., 2018) (Zhou et al., 2004). The protein encoded by Fas is a member of the TNF receptor superfamily. It plays a central role in the physiological regulation of programmed cell death and is associated with various malignancies and immune system diseases. Fas stimulation may contribute to the survival or proliferation of HCC cells (Okano et al., 2003). However, downregulation of Fas expression by HBV might inhibit the apoptosis of HCC cells (Zou et al., 2015).

The remaining genes in the signature are also associated with liver cancer in different ways and play a role in our signature together with these genes in the form of gene pairs. Some of these genes may have a more important effect on the expression imbalance than a single gene with abnormal expression. GSEA was used to analyze the differential expression of genes in the high- and low-risk groups. Consistent with previous reports, the expression of genes related to a number of signaling pathways was significantly different in the high-risk group, including “PI3K-AKT-mTOR signaling” (Zhou et al., 2011; Wang et al., 2017), “DNA_REPAIR” (Lin et al., 2016), “G2M checkpoint” (Yin et al., 2017), and “GLYCOLYSIS” (Qin et al., 2018). In addition, we also found that “UNFOLDED_PROTEIN_RESPONSE”, “E2F_TARGETS”, “MTORC1_signaling” and other hallmarks were also enriched in the high-risk group. As a central tumor suppressor, p53 protects the genome by coordinating multiple DNA damage response (DDR) mechanisms (Williams and Schumacher, 2016). Many mechanisms of DNA repair in cells are influenced by p53. The coordination of DNA repair is an important process through which p53 inhibits tumor development (Janic et al., 2018). It is therefore perhaps unsurprising that the p53 pathway was also enriched in the high-risk group according to the GSEA analysis. Next, we collected the information of patients with P53 mutant HCC from the TCGA data set in the CBioPortal database. We found that P53 mutations were present in 32% of HCC cases, and the risk score of in the mutant group was significantly higher than that in the non-mutant group by calculating the levels of autophagy-related genes. The corresponding results are provided in Supplementary Figure S3. Besides, we found that β-catenin mutations were present in 26% of HCC cases, but we were unable to draw meaningful conclusions by calculating the levels of autophagy-related genes. The corresponding results are provided in Supplementary Figure S4.

In spite of the exciting finding, this study also has several limitations. First, the data were sourced from a limited number of databases, and are not sufficiently broad to prove the universality of the signature. Secondly, the training dataset samples used to establish the autophagy characteristics were derived from previous retrospective studies, and we also need a prospective cohort to verify the results. Prospective studies are needed to further verify the clinical use and biological function of the signature. Future studies will incorporate more datasets and integrate other clinical and pathological indicators, which may provide more useful and accurate results.

## Conclusion

Based on multiple pairs of autophagy-related genes, we proposed a prognostic model for predicting the overall survival of HCC patients after surgery. The gene-air signature is a promising prognostic indicator. The credibility of the model was verified using two unrelated verification sets. Compared with most other existing prognostic models, our model shows a more accurate prediction effect. At the same time, this study further proves the importance of autophagy in the occurrence and development of HCC, and also provides potential therapeutic targets.

## Data Availability

The original contributions presented in the study are included in the article/Supplementary Material, further inquiries can be directed to the corresponding authors.
