# Supplementary figures and images for: Autophagy-Related Gene Pairs Signature for the Prognosis of Hepatocellular Carcinoma
Source: Front Mol Biosci. 2021 May 20;8:670241. doi: 10.3389/fmolb.2021.670241 (PMC8173133; doi:10.3389/fmolb.2021.670241)

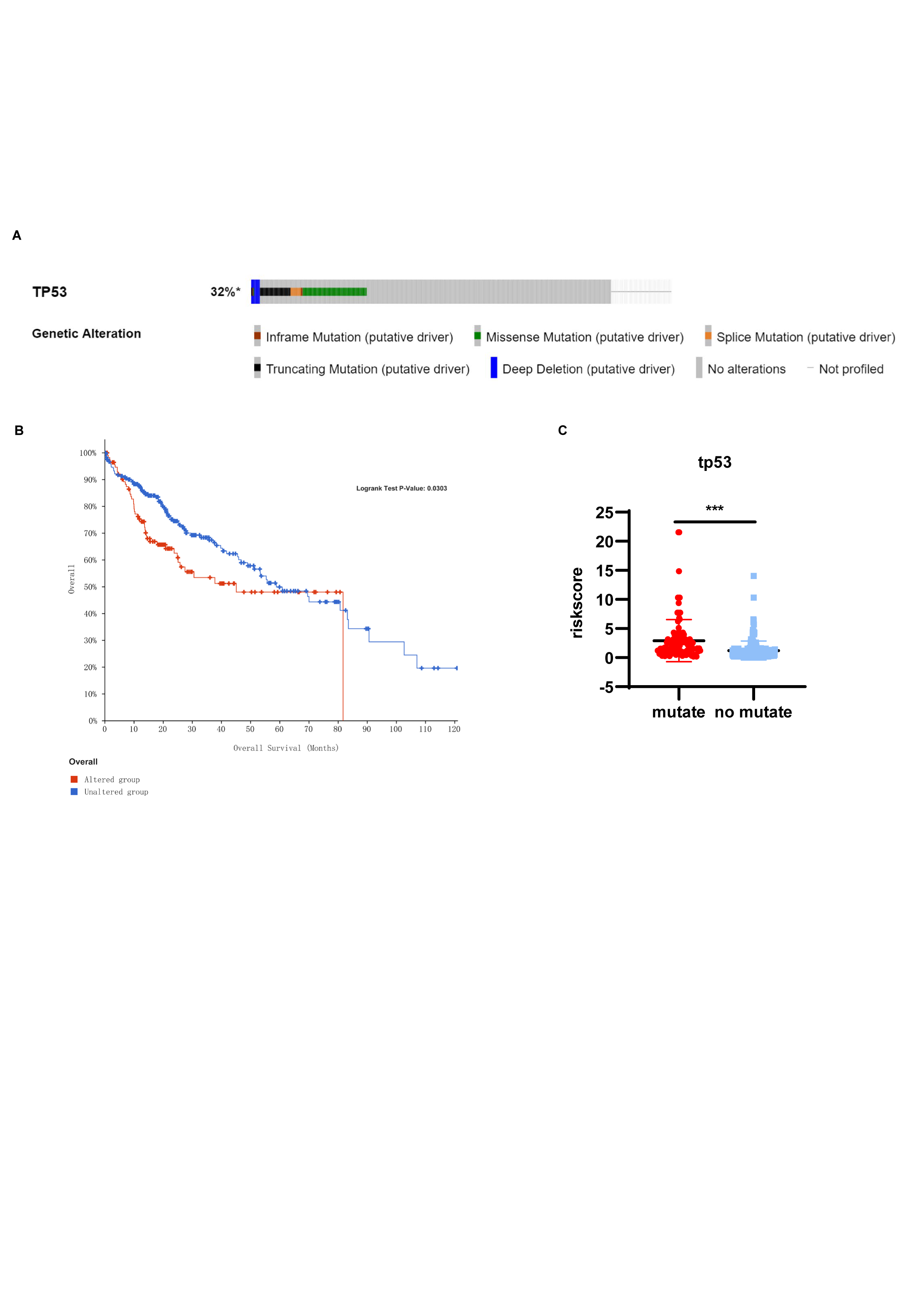

Supplement: Supplementary file 1 [file Image3.TIF]

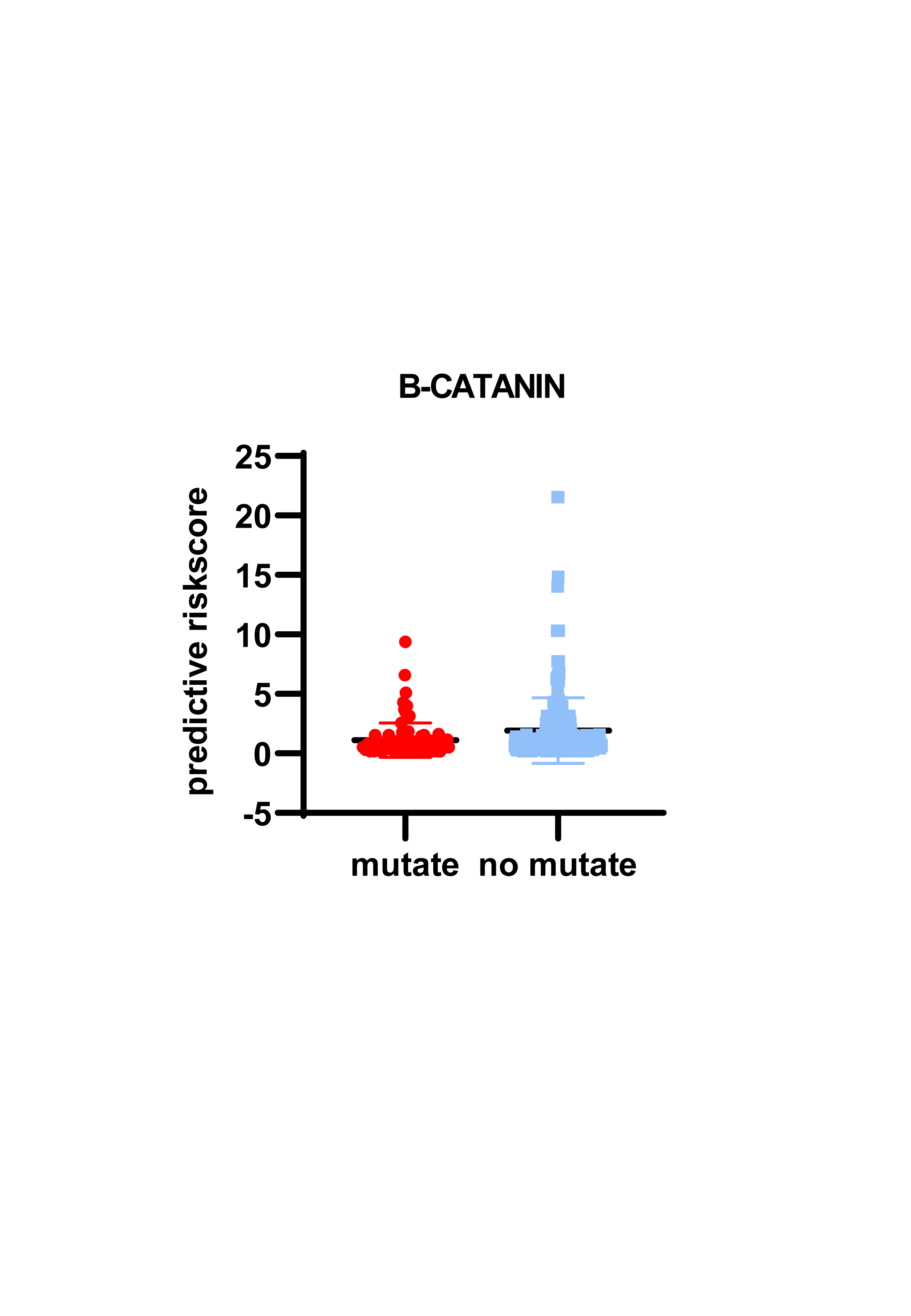

Supplement: Supplementary file 2 [file Image4.TIF]

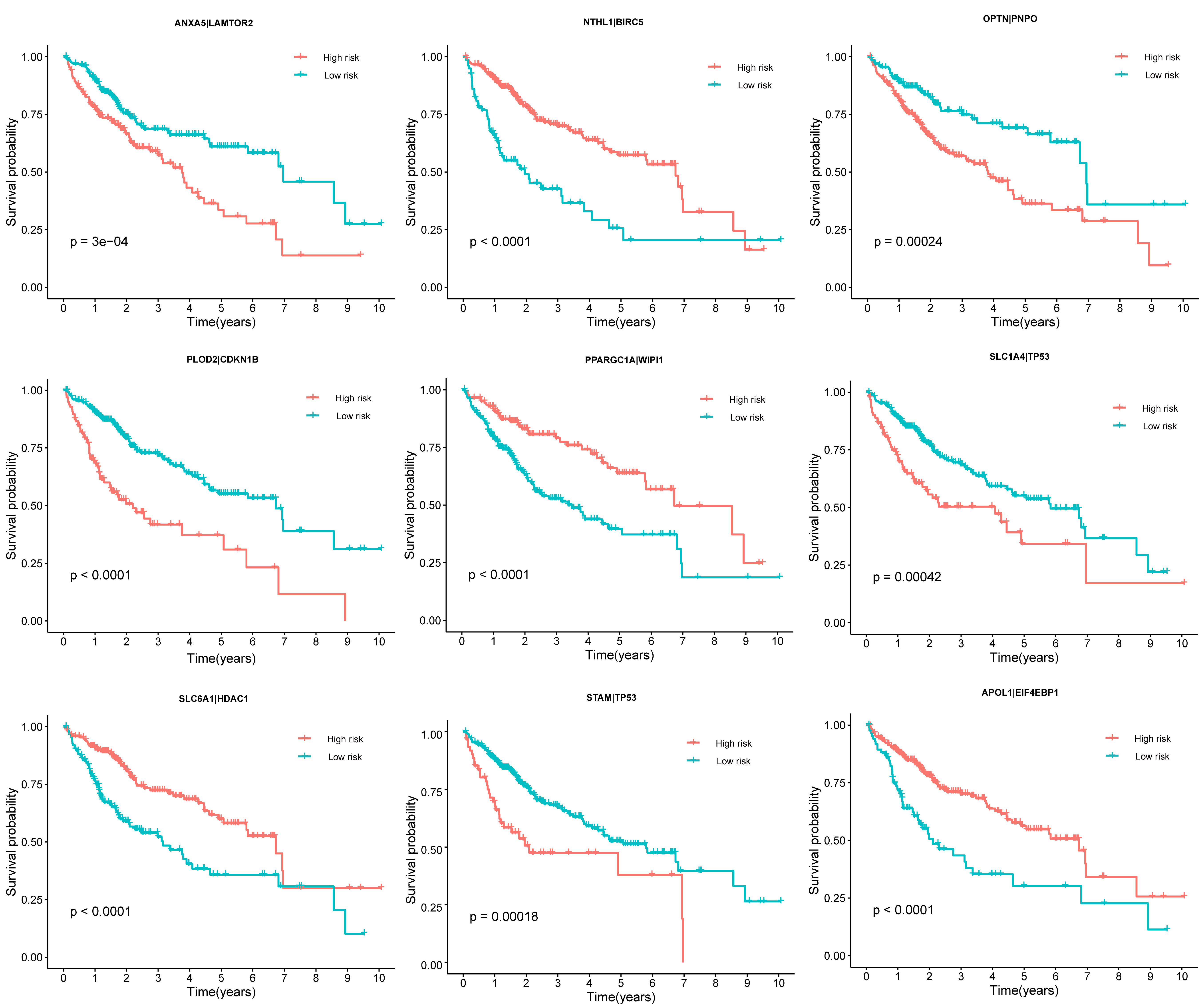

Supplement: Supplementary file 3 [file Image2.TIF]

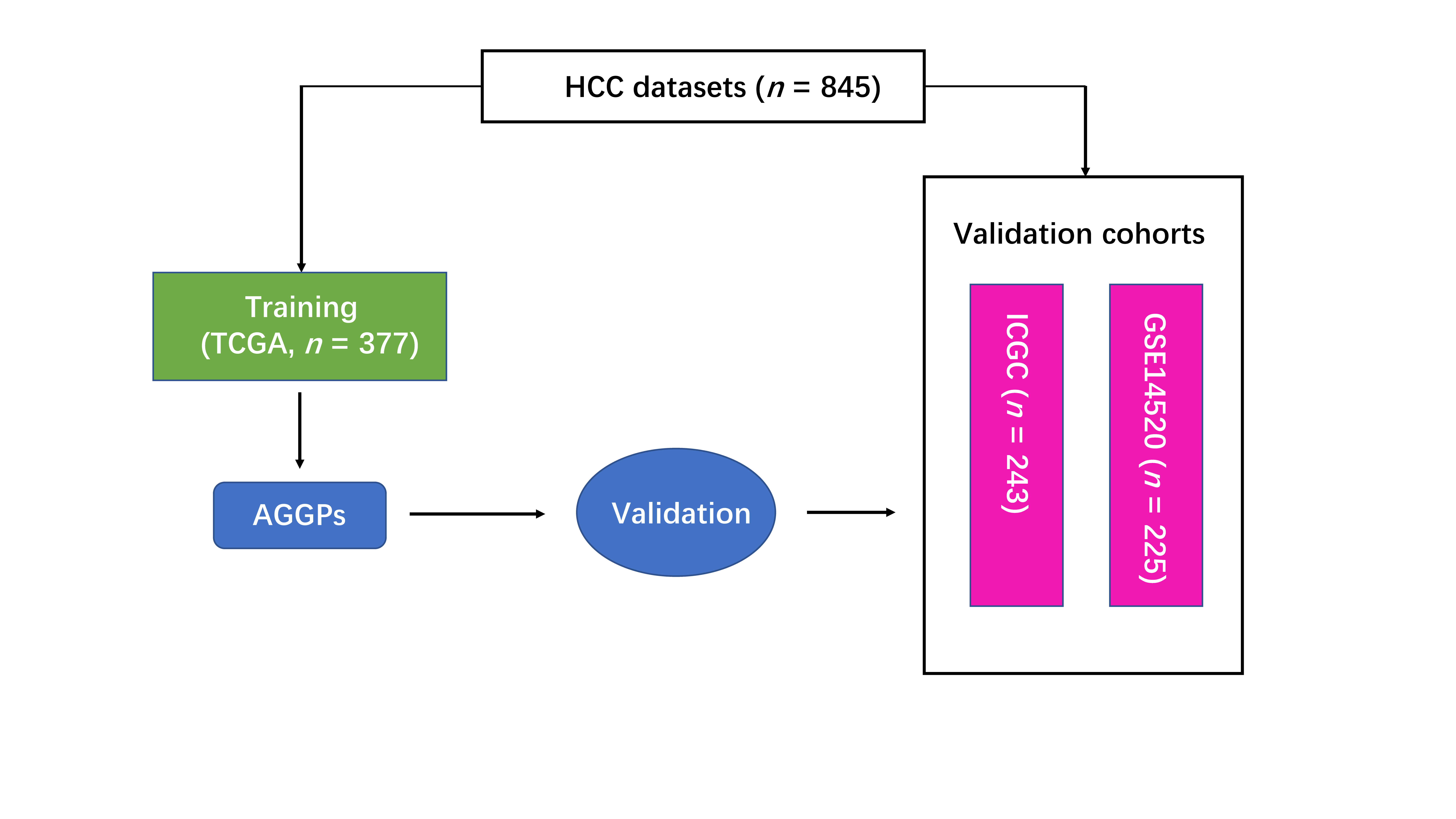

Supplement: Supplementary file 4 [file Image1.TIF]
